# Rapid and consistent genetic clustering of environmental *Legionella pneumophila* isolates using accessible single-step WGS analysis tools

**DOI:** 10.1371/journal.pone.0355944

**Published:** 2026-08-11

**Authors:** Hanno Schmidt, Sven-Ernö Bikár, Bettina Lieb, Daniela Lotz, Tobias Brand, Moritz Brandstetter, Thomas Hankeln, André Michel, Bodo Plachter, Wolfgang Kohnen

**Affiliations:** 1 Sequencing Consortium, University Medical Center of the Johannes Gutenberg-University Mainz, Mainz, Germany; 2 Institute of Virology, University Medical Center of the Johannes Gutenberg-University Mainz, Mainz, Germany; 3 Institute for Quantitative and Computational Biosciences, Johannes Gutenberg-University Mainz, Mainz, Germany; 4 StarSEQ GmbH, Mainz, Germany; 5 Department of Hygiene and Infection Prevention, University Medical Center of the Johannes Gutenberg-University Mainz, Mainz, Germany; 6 Institute of Medical Microbiology and Hygiene, University Medical Center of the Johannes Gutenberg-University Mainz, Mainz, Germany; 7 Institute of Organismal and Molecular Evolutionary Biology, Johannes Gutenberg-University Mainz, Mainz, Germany; 8 Health Care Department, University Medical Center of the Johannes Gutenberg-University Mainz, Mainz, Germany; Universidad San Francisco de Quito, ECUADOR

## Abstract

Genetic characterization of pathogen isolates is increasingly important in healthcare settings, yet the bioinformatic workflows required can be complex and resource-intensive. Here we analyzed 19 environmental *Legionella pneumophila* isolates using whole genome sequencing (WGS) and compared results from an established multi-step bioinformatic pipeline with several accessible, single-step analysis tools. All approaches produced consistent clustering patterns, resolving the isolates into two major genetic clusters. No clear association between spatial sampling distance and genetic relatedness was observed within this dataset: one cluster was detected in two settlements 50 km apart, while both clusters also co-occurred on a single hospital floor. Across this dataset, all WGS-based approaches provided clustering resolution broadly comparable to MLST/cgMLST. These findings indicate that accessible WGS analysis tools can reproduce the main genomic relationships inferred by more complex workflows while reducing analytical complexity and required bioinformatic expertise. Based on these observations, we discuss how rapid low-complexity WGS workflows may support future same-day bacterial isolate characterization in applied settings.

## Introduction

Bacterial strains can be characterized by various molecular genetic methods. Sanger sequencing of the PCR-amplified 16S rRNA gene was the standard method for decades [1]. However, closely related species or strains may have identical or nearly identical 16S sequences and thus be indistinguishable [2]. As a result, neither high-resolution epidemiological classifications nor outbreak analyses at subtype or strain level are possible with 16S rRNA amplicon sequencing. Consequently, the approach was extended to sequencing a defined set of marker genes for better resolution, called multilocus sequence typing (MLST) [3–5]. This procedure is standardized by determining predefined alleles for each target gene, thus creating easily comparable individual strings of allele numbers [6,7]. However, MLST is limited in using only a fraction of all available genetic information, and it has been shown that genetic signatures of different types of genes can result in inaccurate topologies or branch lengths in phylogenetic trees used for classification [8]. Moreover, MLST schemes require the targeted amplification of selected loci of interest, introducing additional sources of error [9]. In recent years, MLST schemes with more loci were designed and nowadays core genome MLST (cgMLST) analyses are making use of up to thousand loci or even more. These analyses require whole-genome sequencing (WGS) of the bacterial isolates. WGS datasets can be used for a broad range of pathogen-typing approaches such as cgMLST, but also techniques that make use of the whole sequence information and not only the defined marker loci. Relevant examples are SNP-based [10] and k-mer-based [11] approaches. All these approaches have fundamentally changed the ability to track microbial genetic patterns [10,12]. Using WGS, close relationships between isolates can be clarified and clinical cases can be linked to environmental samples [13–15]. Notably, the strengths of WGS approaches often go hand in hand with high demands on the implementation of data analysis workflows [16].

*Legionella pneumophila* is the causal agent of Legionnaires’ disease [17], a severe form of pneumonia [18]. The infection is mainly transmitted via the inhalation of aerosols, thus providing a challenge for prevention in healthcare settings. *Legionella* is ubiquitous in water distribution systems [19], with steadily increasing incidences of human infections [20,21]. It has been shown that *L. pneumophila* is genetically and phenotypically structured [22]. In order to understand the distribution of *Legionella* in water distribution systems, and thus the risk of infection, efforts have long been made to decipher the spatial spread of strains, particularly in hospital settings [23,24]. However, the resolution within these studies is highly dependent on the methods used.

In the genetic investigation of *L. pneumophila* outbreaks and water pipe system colonizations, WGS has begun to replace PCR-based typing methods [25], and to replace the MLST schemes that were formerly widely used [26,27]. Recent studies have shown that WGS is capable of exploring the genetic interrelationships even for large datasets of *L. pneumophila* [28,29]. One challenge that currently remains is to make a reliable analysis of samples available to laboratories lacking bioinformatic expertise. Many sequential bioinformatic steps are necessary for processing WGS datasets according to established multi-step bioinformatic workflows, each of which is usually time consuming and resource intensive and requires the handling of various bioinformatics tools and data formats. The aim of the present study was to analyze real world *L. pneumophila* samples with rapid and accessible bioinformatic approaches and to evaluate the consistency of the resulting phylogenetic patterns. To this end, we collected, sequenced, and analyzed environmental *L. pneumophila* samples from three sources: (i) a hospital, (ii) non-hospital buildings in the same city, and (iii) non-hospital buildings in other nearby cities and towns (hereafter referred to as “settlements”). We processed the data with several tools in parallel to compare the resulting clustering patterns generated by user-accessible rapid analysis tools and more complex multi-step workflows. This allowed us to assess whether such simplified approaches could serve as practical complementary approaches for rapid comparative typing analyses between samples. The study was not designed as a formal benchmarking analysis across diverse outbreak scenarios, but rather as an applied comparison of workflow concordance within a real-world environmental dataset. We also illustrate potential implications for the interpretation of *L. pneumophila* distribution patterns and for the hygiene management in complex water distribution systems.

## Materials and methods

Environmental water samples were collected from buildings located in the cities of Mainz (settlement 1), Worms (settlement 2), Ingelheim (settlement 3), and Ockenheim (settlement 4), with specific site details withheld to protect facility privacy. Sampling was carried out under standardized conditions at water taps and faucets that had been disinfected by flame treatment. After flushing 1 liter of water, 250 ml was collected in sterile sampling bottles. The samples were transported in a cooled container and processed within two days. All water samples analyzed in this study were collected as part of routine drinking water monitoring and environmental hygiene investigations conducted under the applicable public health regulations. Sampling from hospital water systems was performed by qualified personnel of the Department of Hygiene and Infection Prevention. Sampling from non-hospital buildings was conducted by the responsible public health authority, the Public Health Office Mainz-Bingen (Gesundheitsamt Mainz-Bingen). All laboratory analyses were carried out in an accredited diagnostic laboratory. As the samples originated from routine regulatory monitoring activities and no additional interventions or sampling were performed, no specific additional permits were required for this study.

Antibiotic-supplemented buffered charcoal yeast extract (BCYE+AB) agar in 10 cm culture dishes served as the growth medium. Two 500 µl aliquots of each sample were pipetted directly onto the agar surface. In addition, 50 ml of each sample was sterile-filtered through a 0.45 μm membrane filter, overlaid with 30 ml of HCl/KCl acid buffer, and rinsed with sterile phosphate-buffered saline (PBS) after five minutes. The treated filter was then placed onto the BCYE+AB plate that already contained the respective untreated aliquots. This procedure ensured that both a small untreated portion – preserving potentially sensitive cells – and a larger, acid-treated and flora-reduced portion – enhancing detection sensitivity – were applied to the culture medium.

Agar plates were incubated at 36 °C under high humidity (achieved by placing a bowl of deionized water in the incubator) for 7–10 days. They were inspected between days 2 and 5 to monitor possible overgrowth by accompanying flora. Colonies showing typical *Legionella*-like morphology (shiny, smooth, gray-white, finely prismatic) were sub-cultured in parallel onto Columbia blood agar and BCYE agar and incubated for an additional 2–5 days at 36 °C with high humidity. Only colonies that grew on BCYE, but not on Columbia blood agar were retained for further analysis.

Species identification was confirmed as *Legionella pneumophila* by MALDI-TOF mass spectrometry using a MALDI Biotyper (Bruker Daltonics, Billerica, MA, USA). Isolates were then suspended in DNA/RNA Shield stabilization solution (Zymo Research Inc., Irvine, CA, USA) and stored until further processing.

DNA isolation was performed on a Maxwell® CSC 48 Instrument (Promega, Madison, USA) in RUO mode using the Maxwell® RSC Blood DNA Kit with modifications according to application note PA-743. The DNA library preparation was done with the NEBNext® UltraExpress™ FS DNA Library Prep Kit (New England Biolabs, Ipswich, USA) according to the manufacturer’s instructions. Sequencing was processed on a NextSeq 2000 instrument (Illumina, San Diego, California, USA) with a P3 or P4 flow cell and XLEAP-SBS chemistry. Paired-end reads of 150 bp length were generated to an approximate 100-fold coverage. Base calling was performed by the NextSeq 1000/2000 Control Software Suite v1.7.1 and the resulting data was converted into FASTQ format using DRAGEN BCL convert version 4.2.7.

Quality processing of raw reads was performed using fastp v.0.20.0 [30], with reads shorter than 30 nucleotides or a q-score below 20 being discarded. Processed reads were mapped to the *Legionella pneumophila subsp. pneumophila* reference genome NC_018140 using BWA v.0.7.17 [31]. Variant calling was performed using freebayes v1.3.2 [32], and subsequent filtering of variants and consensus sequence generation were conducted using bcftools v1.11 [33]. Positions with insufficient coverage or ambiguous base calls were masked as “N” in the final consensus sequence.

All consensus sequences were bioinformatically processed with a dedicated pipeline for the phylogenetic analysis of bacterial genomes: Firstly, the tool Prokka v.1.14.6 [34] with the GenBank file for the *L. pneumophila subsp. pneumophila* reference sequence NZ_CP013742.1 as input was used for individual whole genome annotation of every sample. The output was used for the generation of a concatenated core gene alignment using Panaroo v.1.5.2 [35] with the option ‘-a core’ to include only genes that were present in all samples. This alignment was then analyzed with IQ-TREE v.2.4.0 [36] to build a maximum likelihood-based phylogenetic tree. This pipeline represents an established multi-tool workflow for bacterial genome comparison [37], and is hereafter referred to as the PPI pipeline.

The same genomes were then processed using several bioinformatic tools that allow analysis in a single step: (i) the “Type (Strain) Genome Server” TYGS [38,39] with the setting to restrict query to own data, (ii) the fast core genome alignment and single nucleotide polymorphism (SNP) analysis tool Parsnp v.2.1.3 [40], (iii) the k-mer analysis tool Mashtree v.2.3 [41], and (iv) the algorithm CompareSketch from the BBTools suite v.39.19 [42] for fast whole-genome plus 16S analysis. Unless otherwise stated, all software tools were run using default parameters. Several of these tools have very short processing times due to the underlying algorithms and data management concepts. All resulting phylogenetic trees in Newick format were visualized in IcyTree [43] and rooted using the *L. pneumophila subsp. pneumophila* reference sequence NZ_CP013742.1 (Philadelphia-1 isolate) as the outgroup. Parsnp output was additionally visualized with Gingr v.1.3 [44]. CompareSketch output was visualized in Cytoscape v.3.9.1 [45] applying MCL (Markov Clustering Algorithm) with granularity parameter of 10 in the clusterMaker2 plugin. Multilocus sequence typing (MLST) of the samples was done with legsta v.0.5.1 [46], assigning allele-specific identifiers for the official seven-locus typing scheme curated by the ESGLI/EWGLI MLST database [47]. Core genome MLST (cgMLST) was conducted with the Bruker MBioSEQ Ridom Typer (Bruker Corporation, Billerica, MA, USA) applying default settings.

## Results

We sequenced 19 isolates collected from drinking water systems in four different settlements (towns or villages) within an area of approximately 50 km in diameter in the *Rheinhessen* region of Rhineland-Palatinate, Germany. In settlement 1, one sample was obtained from each of four non-hospital buildings. In addition, the water system of a hospital located in settlement 1 was extensively sampled. Three hospital buildings were included, with one of them examined in greater detail. In this focus building, multiple water outlets were sampled across three wards, each supplied by a separate vertical hot-water line. In the remaining three settlements, one sample each was taken from non-hospital buildings. All isolates were confirmed as *L. pneumophila* by MALDI-TOF prior to sequencing.

In order to make the spatial patterns comprehensible, a custom system was designed to name the samples. Each identifier starts with the settlement (S) the sample originated from, followed by further specifying details, of which only those that are relevant are given: hospital (H), building (B), ward (W), room (R), isolate (I). For example, S1-H-B1-W2-R1-I1 refers to a sample from settlement 1, hospital, building 1, ward 2, room 1, and it is the isolate 1 from this location. The short identifier S2-I1 on the other hand refers to the isolate 1 of settlement 2, where no discrimination of buildings etc. is necessary. Settlement and ward identifiers are highlighted in the figures.

**Fig 1:**
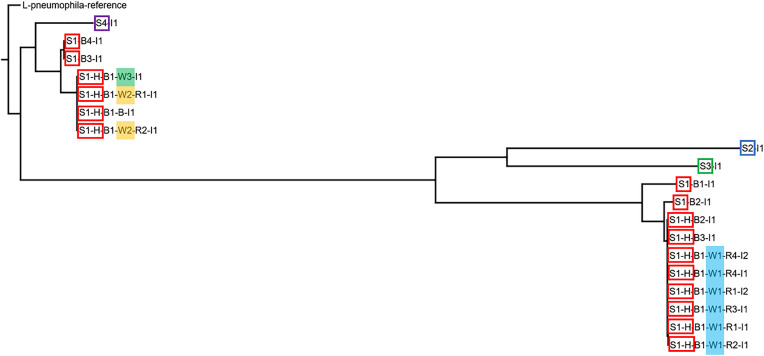
Reference phylogeny from the PPI pipeline. Genome annotation with Prokka, core gene set alignment with Panaroo, and phylogenetic tree generation with IQ-TREE (PPI pipeline). “L. pneumophila reference” refers to NZ_CP013742.1 and is set as the outgroup. Samples are named after the scheme: Settlement (S) – Hospital (H) – Building (B) – Ward (W) – Room (R) – Isolate (I), detailing information of sample origin. Only the information details relevant for unequivocal sample discrimination were used for individual sample names. Settlement (S; or Settlement+Hospital S-H) as the geographically relevant information is highlighted with colored, unfilled boxes. Red boxes represent the city where the focus hospital is located; purple, blue and green boxes represent surrounding settlements. Several wards (W) within one of the hospital buildings were sampled; these are highlighted by colored, filled boxes.

First, we analyzed the genetic relationships between the samples using the PPI pipeline described in the methods section, which assesses an alignment of homologous sequences of all genes present in all individual WGS samples (“core genes”). The PPI pipeline is considered to be a well-established multi-tool workflow for this purpose [37]. The resulting phylogenetic tree shows two distinct clusters comprising all samples (Fig 1). One cluster, hereafter referred to as Cluster A, is quite close to the reference sequence used and includes one non-hospital sample from settlement 4 (S4), two samples from ward 2 (W2), one sample from ward 3 and one sample from the basement (B) of building 1 of the hospital (S1-H-B1), and one each from two other non-hospital buildings of the city where the hospital is located (S1-B3-I1 and S1-B4-I1). The other cluster, hereafter referred to as Cluster B, includes two basal samples with long branches each, one from settlement 2 (S2) and one from settlement 3 (S3). The other samples of cluster B are quite close to each other with two samples from two non-hospital buildings of the city where the hospital is located (S1-B1 and S1-B2), two from other buildings of the hospital (S1-H-B2-I1 and S1-H-B3-I1), and six from ward 1 of the hospital building 1 (S1-H-B1-W1). Especially these six samples from ward 1 are very closely grouped with no visible structuring among them. Thus, both major clusters contain isolates from settlement 1, including samples from the hospital located there. Hence, no clear geographic clustering pattern was apparent within this dataset. At the same time, samples from each ward are strictly confined to one of the two clusters.

Multilocus sequence typing (MLST) with legsta assigned a lineage to 15 out of the 19 samples (Table 1). From cluster A, 5 out of 7 samples were assigned to lineage ST921, including all samples from ward 2 and ward 3. From cluster B, 10 out of 12 samples were assigned to ST1, including all samples from ward 1. The remaining 4 samples (S1-B3-I1, S2-I1, S3-I1, S4-I1) could not be assigned to an established lineage.

**Table 1:** MLST and cgMLST typing. Multilocus sequence typing (MLST) was done with the program legsta that assigns sequence-based types (SBT) based on the allele combination from seven loci. Core genome MLST (cgMLST) was done with the program Ridom Typer that assigns complex type identifiers based on the allele combination from 1,521 loci. Samples are named according to the scheme: Settlement (S) – Hospital (H) – Building (B) – Ward (W) – Room (R) – Isolate (I), detailing information of sample origin. Only the information details relevant for unequivocal sample discrimination were used for individual sample names. Samples are listed in alphabetical order.

Sample	MLST SBT	cgMLST complex type
S1-B1-I1	1	168
S1-B2-I1	1	147
S1-B3-I1	–	828
S1-B4-I1	921	828
S1-H-B1-B-I1	921	1562
S1-H-B1-W1-R1-I1	1	24
S1-H-B1-W1-R1-I2	1	24
S1-H-B1-W1-R2-I1	1	400
S1-H-B1-W1-R3-I1	1	400
S1-H-B1-W1-R4-I1	1	400
S1-H-B1-W1-R4-I2	1	400
S1-H-B1-W2-R1-I1	921	365
S1-H-B1-W2-R2-I1	921	1717
S1-H-B1-W3-I1	921	1718
S1-H-B2-I1	1	1719
S1-H-B3-I1	1	400
S2-I1	–	1720
S3-I1	–	967
S4-I1	–	1721

**Fig 2:**
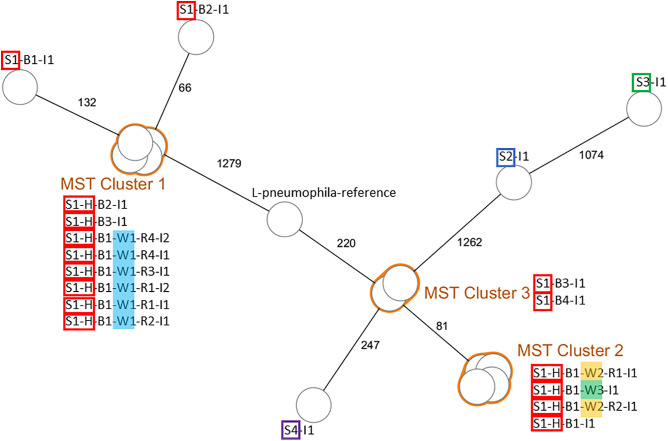
cgMLST network. Core genome MLST analysis with Ridom Typer. Each circle represents one of the 19 samples plus “L-pneumophila-reference” (NZ_CP013742.1). Each node indicates a sample and is labeled accordingly. MST clusters were automatically defined with a distance threshold of four and are highlighted in orange. The lines between sample nodes indicate the closest connection within the network and are labeled according to the number of allele loci differing between the two connected samples. The line lengths between the samples are not proportional to the number of allelic differences. Samples are named after the scheme: Settlement (S) – Hospital (H) – Building (B) – Ward (W) – Room (R) – Isolate (I), detailing information of sample origin. Only the information details relevant for unequivocal sample discrimination were used for individual sample names. Settlement (S; or Settlement+Hospital S-H) as the geographically relevant information is highlighted with colored, unfilled boxes. Red boxes represent the city where the focus hospital is located; purple, blue and green boxes represent surrounding settlements. Several wards (W) within one of the hospital buildings were sampled; these are highlighted by colored, filled boxes.

Core genome MLST (cgMLST) analysis with the commercial Ridom Typer software further split the MLST lineages (Table 1). Every sample is assigned to a “complex type” based on the alleles from 1,521 loci. The five samples that were assigned to lineage ST921 by MLST were assigned to five different “complex types” by cgMLST. The ten samples that were assigned to lineage ST1 by MLST were assigned to five different complex types by cgMLST, with five of them being assigned to complex type 400. All samples were assigned to a complex type. However, five samples were individually assigned to complex types newly established for these samples (S1-H-B1-W2-R2-I1, S1-H-B1-W3-I1, S1-H-B2-I1, S2-I1, S4-I1). Three clusters were established based on the differences between samples with a cluster distance threshold of four (Fig 2). The samples that formed cluster A in the PPI pipeline analysis are split into MST clusters 2 and 3 plus one adjacent sample (S4-I1), and the samples that formed cluster B in the PPI pipeline analysis are grouped in MST cluster 1 plus two adjacent samples (S1-B1-I1, S1-B2-I1). Two samples that formed a distinct clade that grouped with cluster B in the PPI pipeline analysis are now closer to MST cluster 3.

In an alternative approach, we analyzed our data with the online server TYGS for prokaryote taxonomy based on the Genome-to-Genome Distance Calculator [48] and the LPSN database [49]. Using this web tool does not require any bioinformatic knowledge, but it also does not allow deeper insight into the processes or any parameter control. Overall, the results generated here (S1 Fig) are largely consistent with the topology of the PPI pipeline reconstruction depicted in Fig 1. The two major clusters consist of the very same samples and show similar branch lengths. One minor difference is visible within the six samples from ward 1 in cluster B. Here, two samples (S1-H-B1-W1-R1-I1 and S1-H-B1-W1-R2-I1) are grouped and stick out from the rest.

**Fig 3:**
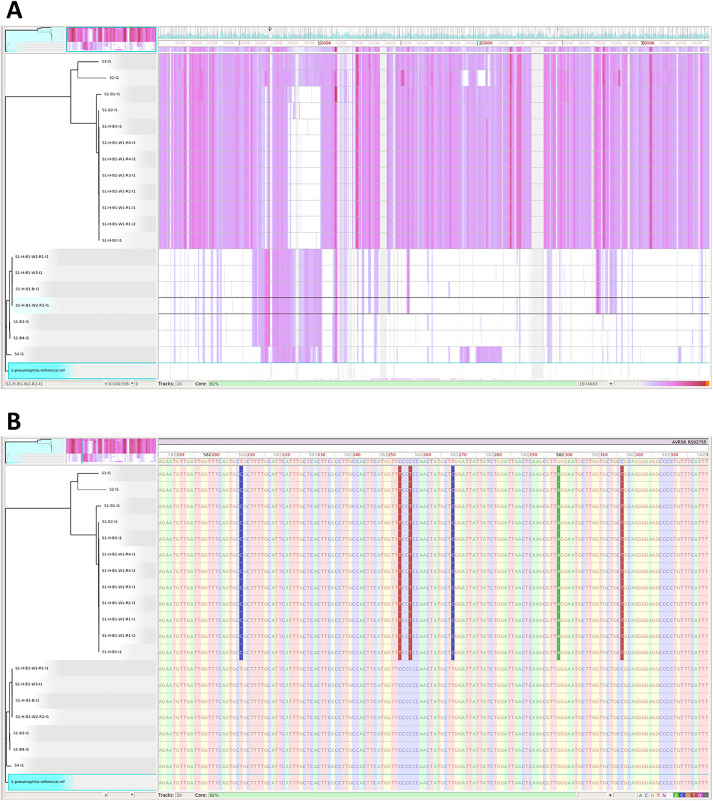
Detailed visualization of Parsnp output with Gingr. The alignment generated with Parsnp is displayed alongside the calculated phylogenetic tree, making the SNP conformation across the samples visible. The upper samples make up cluster B, the lower samples make up cluster A. (A) The full alignment with broader patterns of genetic differentiation between the genetic clusters A and B. Pink positions highlight differences of the sample in relation to the reference sequence. The two genetic clusters A and B are easily distinguishable by shared SNPs and a clear separation between them. (B) A zoom into the alignment to illustrate the clear separation of the genetic clusters over multiple SNPs in a row. The individual SNPs shown are present in all samples of cluster B and in none of cluster A.

As a third approach, the phylogeny was generated by the command-line tool Parsnp (S2 Fig), again producing a highly similar result to the one generated by the PPI pipeline. The topology showed no major topological differences and branch lengths differed only marginally. Additionally, the Parsnp output was visualized using the program Gingr. The two well-separated clusters are strikingly reflected in the divergent SNP patterns across the genome (Fig 3).

A fourth analytical approach, using the MinHash value similarity comparison program Mashtree, again produced highly similar clustering patterns as the PPI pipeline (S3 Fig). The topology obtained with both methods was largely identical, branch lengths were similar. Interestingly, the result was even closer to the one obtained from the web resource TYGS (S1 Fig) with the two samples from ward 1 forming a distinct branch within the other ward 1 samples, as described above.

**Fig 4:**
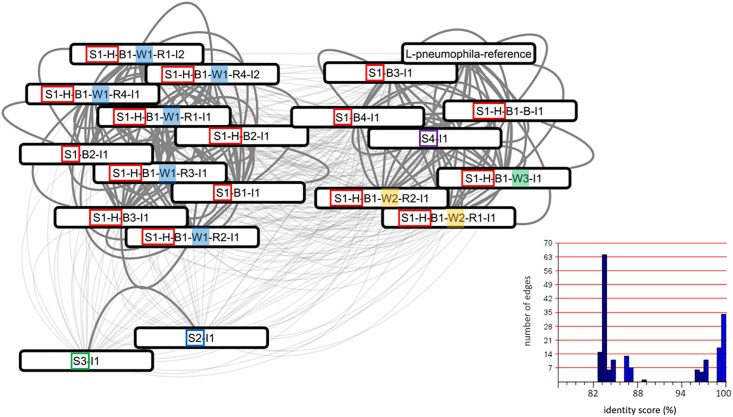
Genetic clustering of samples. Genetic clustering was done by fast whole genome plus 16S all-versus-all comparison with CompareSketch from BBTools. Edge lengths and breadths between nodes reflect relative genetic similarity between the two samples compared. A few nodes have been minimally moved to improve readability. “L. pneumophila reference” refers to NZ_CP013742.1. Samples are named after the scheme: Settlement (S) – Hospital (H) – Building (B) – Ward (W) – Room (R) – Isolate (I), detailing information of sample origin. Only the information details relevant for unequivocal sample discrimination were used for individual sample names. Settlement (S; or Settlement+Hospital S-H) as the geographically relevant information is highlighted with colored, unfilled boxes. Red boxes represent the city where the focus hospital is located; purple, blue and green boxes represent surrounding settlements. Several wards (W) within one of the hospital buildings were sampled; these are highlighted by colored, filled boxes. The histogram in the lower right corner shows the distribution of pairwise identity scores for the all-versus-all comparison.

An operationally distinct, yet comparable result output was generated using CompareSketch from the BBTools suite. This tool calculates pairwise genomic distances, which were visualized as a network. Although the representation differs from tree-based methods, the resulting structure closely mirrored the previously obtained phylogenies (Fig 4). Two well-separated clusters were again apparent, containing the same sets of samples as in the analyses described above. The reference sequence and the individual samples from settlements 2 and 3 also occupied consistent positions relative to the other isolates. We also used the scores for average nucleotide identity, exact identity of the 16S, and percent identity in k-mer space obtained by CompareSketch for a tabular analysis alongside the graphical representation. We calculated an average of the three scores to generate an overall identity score. This composite identity score was used as a heuristic measure to facilitate qualitative comparison between samples and should not be interpreted as a validated standardized metric. The pairwise comparisons of samples produced a clear and consistent separation of the two major clusters, with all comparisons among samples within the same cluster yielding scores ≥ 99.5 and all comparisons between samples from different clusters yielding lower scores. While this observation may indicate potential utility for rapid qualitative sample assignment within this dataset, the proposed threshold was not independently validated and should therefore be interpreted cautiously.

The present analyses were primarily qualitative and were not designed to systematically evaluate fine-scale phylogenetic resolution or outbreak-level discrimination. Consequently, minor topological differences between closely related isolates should be interpreted cautiously.

All analyses, except those performed with TYGS, were conducted on a desktop computer running Ubuntu 22.04 within a Windows Subsystem for Linux (WSL) environment. The number of threads used was controlled manually for all bioinformatic steps by setting the respective program parameters. For our dataset, the PPI pipeline took 54 minutes on four parallel computing threads without the data handling in between steps. By increasing the number of parallel computing threads to 50, this duration could only be reduced by 9.3%, which means that the potential time savings probably remain very limited even in a high-performance computing environment. In our case, the analysis using the online server TYGS took 36 minutes, but this can vary depending on the number of requests, which limits influence on the runtime. The web resource also limits the analysis to a maximum of 50 samples. The programs Parsnp, Mashtree, and CompareSketch all needed less than two minutes on four computing threads to produce final results from the sequence data for all 19 samples in parallel. This corresponds to a time saving for the bioinformatic analysis post mapping/assembly of approximately 96% relative to the PPI pipeline in our specific setting and facilitates rapid workflow execution.

## Discussion

Here we present a real-world example of a whole genome sequencing-based typing analysis of *Legionella pneumophila* samples with bioinformatic tools of varying complexity. We show that results produced by five different approaches showed largely concordant clustering patterns without requiring extensive bioinformatic expertise and resources. Several of the investigated tools enabled rapid initial analyses with minimal interaction on a standard desktop computer (four CPU threads in our setting). The study was motivated by the need for rapid responses to diagnostic inquiries and by the possibility of enabling analyses by non-specialist personnel.

This study has several limitations. The analysis was based on a limited number of environmental isolates collected within a relatively restricted geographic region and was not designed as a comprehensive benchmarking study across broad genomic diversity or outbreak scenarios. Consequently, the findings should be interpreted as an applied evaluation of workflow concordance within this specific dataset rather than as a universal performance assessment of the investigated tools. No independent epidemiological ground truth or confirmed outbreak structure was available for the investigated isolates. Consequently, concordance between methods should not be interpreted as direct evidence of phylogenetic accuracy but rather as agreement between analytical approaches within this dataset. Future studies including larger and geographically more diverse isolate collections will be necessary to systematically assess the generalizability of the observed concordance patterns.

### Rapid genetic clustering

Across all analyses we consistently received two genetic clusters. According to multilocus sequence typing (MLST), cluster A mainly consists of *L. pneumophila* lineage ST921, and cluster B mainly of ST1. The reference sequence used in this study clusters with the ST921 sequences, which is plausible because it belongs to lineage ST36 (own analysis, and see [50]) rather than ST1, as is the case for several other published *L. pneumophila* reference genomes. Notably, 4 out of the 19 (21%) sequences could not be assigned to a lineage by MLST. This illustrates a limitation of the commonly used method based on a well-characterized but small set of marker genes, as only samples can be typed that show a coherent set of alleles [51]. Also, the MLST approach was only suitable to assign the samples to one of the two sequence types (ST’s) but provided no further resolution among closely related isolates (Table 1). A significant increase in the number of marker genes (core genome MLST, cgMLST) mitigates these limitations largely [27], but the inherent restrictions of MLST-style schemes remain [52]. Moreover, cgMLST implementations are often commercial and costly (e.g., the Ridom Typer used here), or they again demand considerable bioinformatic expertise (e.g., chewBBACA [53] or pyMLST [54]). The cgMLST results expectably showed a higher level of discrimination than the MLST results. The two lineages ST1 and ST921 that comprised most of the sequences in the MLST analysis were split into ten complex types in the cgMLST analysis. While this increased resolution improves discrimination between closely related isolates, it also complicates direct comparison with broader lineage-level classifications. Moreover, the sequence types produced by legsta follow the globally standardized MLST nomenclature, whereas the complex types generated by Ridom Typer are proprietary identifiers that cannot be readily integrated with other typing schemes. The cgMLST network representation largely reflected the results generated with the PPI pipeline (Fig 1 and Fig 2). However, cluster A was subdivided into two MST clusters and several non-hospital samples were connected in a way that did not reflect the topology of the other WGS analyses including the PPI pipeline (see results above). Overall, the MLST and cgMLST analyses showed useful results with practical challenges each.

Using a set of whole genome analysis methods which represent an alternative to the marker gene-based (cg)MLST approaches, we were able to consistently position all samples within the inferred clustering structures. Moreover, the generated phylogenetic trees (Fig 1, S1-S3 Fig) and the network representation (Fig 4) gave additional information, e.g., the structure within cluster B. Here, all samples from ward 1 were closely related and clustered together with two additional samples from other buildings of the same hospital (S1-H-B3-I1, S1-H-B2-I1). Also, two samples from outside the hospital but from the same city (S1-B2-I1, S1-B1-I1) showed close genetic similarity to these nearly identical isolates from the hospital, while forming slightly separated branches. These observations illustrate that rapid WGS analysis tools can preserve minor clustering differences between closely related isolates within this dataset.

Minor topological differences between the cgMLST analysis and the other investigated approaches are likely attributable to methodological differences between allele-based typing schemes and alignment- or SNP-based phylogenomic approaches. In contrast to core genome alignment methods, cgMLST relies on predefined allele schemes and discrete allele assignments, which may affect clustering behavior in closely related isolates. Such differences are expected and do not necessarily indicate incorrect clustering by either approach.

### Implications for spatial lineage distribution and hygiene management

Interestingly, no clear association between geographic proximity and genetic similarity was observed within this dataset. Samples from settlement 1 – and even samples from a single floor of one hospital building there – were assigned to both *L. pneumophila* clusters. On the other hand, several samples from non-hospital buildings of the settlement were relatively closely related to hospital samples from each cluster. Despite being the most geographically distant pair, settlements 2 and 3 consistently cluster together in all analyses, albeit with long individual branches. All analyses combined suggest that the area under investigation may be populated by two main lineages of *L. pneumophila* (cluster A and cluster B) which do not appear to have clear geographical boundaries. This illustrates limitations of classical MLST analyses in resolving relationships among closely related isolates. For our dataset we could reproduce the two clusters with MLST: all samples that could be typed were assigned to either ST1 or ST921. However, besides the fact that 21% of samples could not be typed, no additional information on relatedness between samples could be gained by MLST analyses. In contrast, the WGS analyses preserved additional clustering structure among closely related isolates. For example, samples from non-hospital and hospital samples of settlement 1 could still be differentiated by WGS although they were assigned to the same MLST identifier. In contrast, the cgMLST analysis produced a higher degree of subdivision, assigning nine samples to private complex types that were not shared with any other sample.

**Fig 5:**
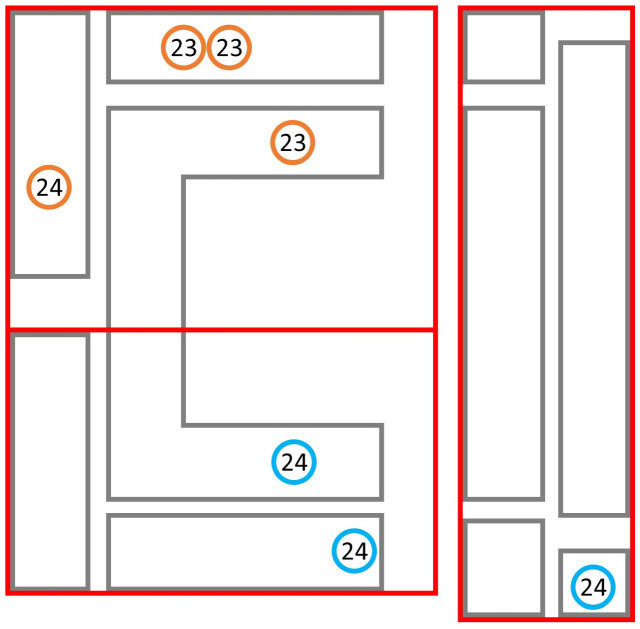
Schematic location map from within the hospital where the samples were taken. The gray shapes indicate the areas with rooms (mostly equipped with patient beds). The three red rectangles indicate the areas that are served by separated water supply piping. These areas also correspond to the wards monitored in this study (W1 = top, W2 = bottom, W3 = right). The circles indicate the sampling locations, where circle colors represent the genetic cluster to which they belong (blue = cluster A, orange = cluster B), and the number in the middle indicates the year of sampling (23 = 2023, 24 = 2024).

A particularly noteworthy case is the relationship among samples obtained from the different wards of hospital building 1. Each ward is supplied by its own vertical hot-water line, and, despite their extreme spatial proximity, we observed the presence of both distinct genetic lineages. All samples from ward 2 and ward 3 were assigned to cluster A, and samples from ward 1 were exclusively assigned to cluster B (Fig 5). This finding is of particular relevance in view of the infection control challenges associated with *L. pneumophila*. Understanding such distribution patterns may be useful to help inform the planning, maintenance, and monitoring of hot-water systems, thereby contributing to improved patient safety.

### Towards same-day genomic typing

The analysis of WGS data is often complex and therefore usually requires genome experts to run elaborate bioinformatics pipelines. However, personnel skilled in such analyses is frequently unavailable in diagnostic laboratories, and time constraints make these procedures even less attractive. We tested whether tools that require minimal user interaction and bioinformatic expertise for application generate comparable results to those of an established multi-tool pipeline for a set of real-world samples. In our case these one-tool-solutions reduced computing times for bioinformatic analyses post mapping/assembly by 96%. The resultant topologies of the phylogenetic analyses did not show major topological differences between the approaches used. All analyses resulted in two major genetic clusters and all samples were assigned to the same cluster in every analysis. While the trees showed high overall concordance, the methods differed substantially in terms of handling and run times.

**Fig 6:**
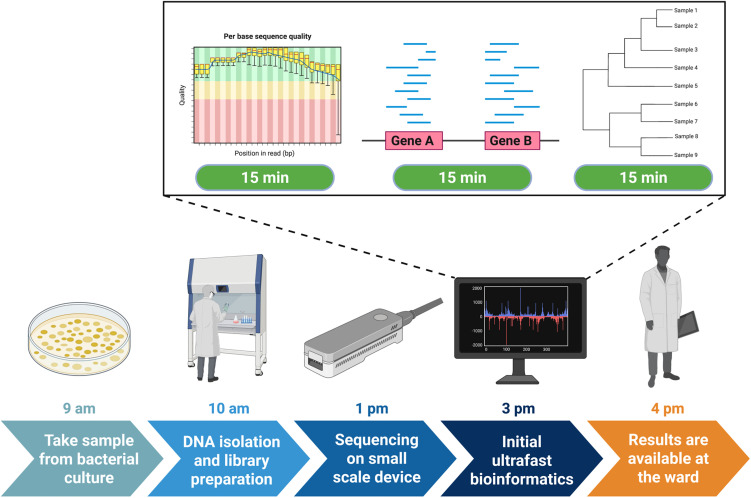
Outline for a potential same-day workflow from sample to result using rapid bioinformatic analyses. This exemplary workflow combines rapid sequencing and ultrafast downstream bioinformatic analyses for low-throughput bacterial isolate characterization. The estimated time intervals are provided as illustrative examples only and were not experimentally validated in this study. The sequencing data analyzed in this work were generated using Illumina sequencing. Practical implementation of such a workflow would depend on sequencing technology, data quality, laboratory organization, and available computational infrastructure. Illustration created in BioRender. Schmidt, H. (2026) https://BioRender.com/260pwxq.

In our current working scheme, there is still room for further development and adaptation to site-specific conditions, with the potential to simplify the overall workflow. For example, using the portable long-read sequencer MinION (Oxford Nanopore Technologies, Oxford, UK) could further reduce operational barriers and sample turnaround times. Combined with automated downstream analyses, the rapid bioinformatic approaches evaluated in this study may enable a conceptual same-day workflow for urgent low-throughput applications (Fig 6). However, such a workflow was not experimentally implemented in this study and would depend on factors including sequencing technology, data quality, assembly quality, and local computational infrastructure. The reduced analytical complexity of the evaluated tools may nevertheless facilitate broader implementation of WGS-based bacterial typing in routine diagnostics and infection control settings.

## Supporting information

S1 Fig Phylogeny from web resource TYGS. Tree generated with the type strain genome server (https://tygs.dsmz.de). “L. pneumophila reference” refers to NZ_CP013742.1 and is set as the outgroup. Samples are named after the scheme: Settlement (S) – Hospital (H) – Building (B) – Ward (W) – Room (R) – Isolate (I), detailing information of sample origin. Only the information details relevant for unequivocal sample discrimination were used for individual sample names. Settlement (S; or Settlement+Hospital S-H) as the geographically relevant information is highlighted with colored, unfilled boxes. Red boxes represent the city where the focus hospital is located; purple, blue and green boxes represent surrounding settlements. Several wards (W) within one of the hospital buildings were sampled; these are highlighted by colored, filled boxes.
(TIF)

S2 Fig Phylogeny from Parsnp. Fast alignment of core genome components and subsequent SNP analysis with the program Parsnp. “L. pneumophila reference” refers to NZ_CP013742.1 and is set as the outgroup. Samples are named after the scheme: Settlement (S) – Hospital (H) – Building (B) – Ward (W) – Room (R) – Isolate (I), detailing information of sample origin. Only the information details relevant for unequivocal sample discrimination were used for individual sample names. Settlement (S; or Settlement+Hospital S-H) as the geographically relevant information is highlighted with colored, unfilled boxes. Red boxes represent the city where the focus hospital is located; purple, blue and green boxes represent surrounding settlements. Several wards (W) within one of the hospital buildings were sampled; these are highlighted by colored, filled boxes.
(TIF)

S3 Fig Phylogeny from Mashtree. Tree generated on basis of k-mer analysis with Mashtree. “L. pneumophila reference” refers to NZ_CP013742.1 and is set as the outgroup. Samples are named after the scheme: Settlement (S) – Hospital (H) – Building (B) – Ward (W) – Room (R) – Isolate (I), detailing information of sample origin. Only the information details relevant for unequivocal sample discrimination were used for individual sample names. Settlement (S; or Settlement+Hospital S-H) as the geographically relevant information is highlighted with colored, unfilled boxes. Red boxes represent the city where the focus hospital is located; purple, blue and green boxes represent surrounding settlements. Several wards (W) within one of the hospital buildings were sampled; these are highlighted by colored, filled boxes.
(TIF)
